# Oral health effects of tobacco and e-cigarettes in Madinah

**DOI:** 10.3389/froh.2025.1698579

**Published:** 2025-11-27

**Authors:** Ahmed M. Kabli, Rawan K. Kamal, Ahmad A. Othman, Fatimah M. Almehmadi, Shuruq A. Alrehaili, Alanoud S. Almurowbae, Rahma F. Alhazmi, Sarah B. Alrashidi, Mahir A. Mirah

**Affiliations:** 1 Department of Preventive Dental Sciences, College of Dentistry, Taibah University, Madinah, Saudi Arabia; 2 Department of Oral and Maxillofacial Diagnostic Sciences, College of Dentistry, Taibah University, Madinah, Saudi Arabia; 3 Internship Program, College of Dentistry, Taibah University, Madinah, Saudi Arabia; 4 College of Dentistry, King Abdulaziz University, Jeddah, Saudi Arabia; 5 RAM Clinics, Yanbu Al-Bandar, Saudi Arabia; 6 Department of Restorative Dental Science, College of Dentistry, Taibah University, Medinah, Saudi Arabia

**Keywords:** periodontal disease, halitosis, nicotine, perceptions, awareness, Saudi Arabia

## Abstract

**Introduction:**

Tobacco smoking and the use of electronic cigarettes (e-cigarettes) are well known to harm oral health, but the dangers of e-cigarettes are still not fully acknowledged, especially in Saudi Arabia where their use is steadily increasing. This study explored awareness of the oral and dental health consequences of tobacco and e-cigarette use among residents of the Al-Madinah region and compared awareness levels across smokers, e-cigarette users, dual users, and non-smokers.

**Methods:**

A cross-sectional online survey was carried out on 278 participants who are 16 years and over in 2025. A validated self-administered questionnaire was used in the collection of data on demographic characteristics, smoking habits, oral health habits, and awareness of smoking-related oral health risks. Chi-square and Fisher Exact tests were used to analyze the data at a statistical significance level of *p* ≤ 0.05.

**Result:**

Of the respondents, 6.8% were traditional smokers, 6.5% were e-cigarette users, 2.2% were dual users and 84.5% were non-smokers. Although more than 80% identified smoking as a cause of oral health problems such as cancer, discoloration, halitosis, and impaired healing, only 17.6% considered e-cigarettes as harmful. The non-smokers and cigarette smokers were more aware than the e-cigarette and dual users who were more likely to underestimate the risks associated with e-cigarettes.

**Conclusion:**

The results indicate that although the level of awareness on the negative effect of tobacco is high, the misconceptions on e-cigarettes are still present, thus suggesting the need for a public health campaign.

## 1 Introduction

Tobacco use remains a major global public health issue, contributing to substantial morbidity and mortality worldwide. It accounts for millions of preventable deaths each year and is recognized by the World Health Organization (WHO) as a leading cause of 8.2 million death (1–3). In Saudi Arabia, around 3.35 million adults aged 15 and above continue to use tobacco products (4). This highlights the critical need for effective prevention and intervention strategies (3).

Tobacco use is strongly associated with lung, head, and neck cancers, alongside a wide range of oral health issues (5). In recent years, electronic nicotine delivery systems (ENDS), or e-cigarettes, have gained popularity as perceived safer alternatives to traditional smoking (6, 7).

First developed in 2003 by Chinese pharmacist Hon Lik, their use expanded rapidly with improved designs, aggressive marketing, and appealing flavors, especially among youth and young adults (8, 9). E-cigarettes come in various forms and nicotine concentrations, with most containing e-liquids made of flavorings, solvents, and nicotine (6). Though marketed as a safer alternative, the inhaled aerosol contains harmful substances, including ultrafine particles, heavy metals, and toxic chemicals that can cause oxidative stress and inflammation in oral tissues (10). Reported oral health effects include dry mouth, mucosal irritation, ulcers, palatal burns, and periodontitis (11), with severe cases involving palatal and respiratory tract injuries (4). Additionally, vaping may disrupt the oral microbiome, increasing the risk of caries and periodontal disease (12). Despite perceptions of e-cigarettes as less harmful, evidence indicates they pose both shared and unique oral health risks compared to traditional smoking (11, 13, 14).

In Saudi Arabia, especially among younger individuals, awareness of the oral and general health risks of tobacco and e-cigarette use remains limited (7, 15, 16). Therefore, this study aimed to evaluate the level of awareness regarding the oral and dental health effects of tobacco and e-cigarette use among residents of the Al-Madinah region and to compare awareness levels between smokers, e-cigarette users, dual users, and non-smokers.

## 2 Methods

### 2.1 Study design and setting

A cross-sectional, online survey-based study was conducted among the Saudi population residing in the Al-Madinah region, Kingdom of Saudi Arabia. The study was designed to assess self-reported adverse effects of electronic cigarettes and traditional tobacco smoking on oral and dental health.

### 2.2 Study population and sampling

All individuals aged 16 years and above, currently living in the Al-Madinah region, were invited to participate in the study. The inclusion criteria encompassed both smokers and non-smokers, regardless of gender or nationality, as long as they resided in the Al-Madinah region and consented to participate.

A non-probability convenience sampling technique was used to recruit participants through electronic means. The survey was distributed via multiple online platforms, including social media applications such as WhatsApp, Facebook groups, and emails, targeting community groups and networks within the Al-Madinah region to maximize reach and participation.

The required sample size was calculated using Cochran's formula for sample size estimation with finite population correction. According to the General Authority for Statistics (17), the total population of the Al-Madinah region was 2,137,983 (18). Assuming a 95% confidence level, a 10% margin of error, and an anticipated response distribution of 50%, the minimum required sample size was calculated to be 196 participants. To enhance representativeness and account for potential incomplete or invalid responses, a total of 278 completed questionnaires were included in the final analysis.

### 2.3 Data collection

An electronic questionnaire, developed using Google Forms, was distributed to the target population. The survey commenced with a clear, introductory statement outlining the study's purpose, the voluntary nature of participation, and assurances of confidentiality. It emphasized that participation was anonymous, and no personal identifying information such as names, email addresses, or contact details would be collected. Participants were informed that they could complete the questionnaire once, with the option to review and modify their responses prior to final submission.

The questionnaire was self-reported and collected using an online survey; therefore, the study may be at risk of recall bias and social desirability bias. To help limit these biases, we designed the survey to be anonymous, based on voluntary participation, and built on a previously validated questionnaire.

The questionnaire used in this study was adapted from previously validated instruments applied in similar previous research (1, 6, 16, 19–22), and was designed to collect comprehensive information through closed-ended multiple-choice questions. It comprised several key sections. The first section gathered sociodemographic data, including participants' age, gender, and educational level. The second section assessed participants' tobacco and electronic cigarette use, focusing on the type of product used, frequency of use, and duration of the habit. The third section explored oral hygiene practices, covering the frequency of tooth brushing, use of mouthwash, interdental cleaning aids, and routine dental visits. The fourth section examined participants' self-perceived oral and dental symptoms associated with smoking, including perceived changes in gum health, tooth discoloration, bad breath, and the occurrence of oral lesions.

### 2.4 Statistical analysis

Data were analyzed using SPSS version 22.0 (SPSS Inc., Chicago, IL). Descriptive statistics summarized demographic characteristics and participants' questionnaire responses as frequencies and percentages. The Chi-square test was used to compare categorical variables between smoker categories (traditional, electronic, both, and non-smokers). Fisher's Exact Test was applied when expected counts were low. A *p*-value of <0.05 was considered statistically significant.

### 2.5 Ethical considerations

The study adhered to the Declaration of Helsinki principles and received approval from the Research Ethics Committee of Taibah University, Al-Madinah, Saudi Arabia with protocol number TUCDREC/060324/AKabli. Participation was voluntary, with electronic informed consent obtained from all respondents. The questionnaire assured anonymity, confidentiality, and the right to withdraw without consequence. No personal identifiers were collected, and data were securely stored for research use only. This study was conducted in adherence to the Strengthening the Reporting of Observational Studies in Epidemiology (STROBE) guidelines.

## 3 Results

The study analyzed data from 278 Saudi residents living in the Al-Madinah region, Saudi Arabia. Table 1 presents the sociodemographic characteristics of the 278 participants included in the study. Most respondents were young adults aged 21–30 years, accounting for 51.1% of the sample, followed by those aged 16–20 years (22.3%) and 31–50 years (23.4%). Only a small proportion (3.2%) were older than 50 years, indicating that the study population was predominantly composed of younger individuals. In terms of sex distribution, most participants were female (77.3%), while males constituted 22.7% of the sample. Regarding educational attainment, 69.1% of the participants had achieved a university-level education or higher, while 30.9% had a secondary school education or less.

**Table 1:** Characteristics of the studied participants (*n* = 278).

Characteristics	*n* (%)
Age in years	
16–20	62 (22.3)
21–30	142 (51.1)
31–50	65 (23.4)
>50	9 (3.2)
Sex	
Male	63 (22.7)
Female	215 (77.3)
Educational level	
Secondary and lower	86 (30.9)
University and higher	192 (69.1)

**Figure 1:**
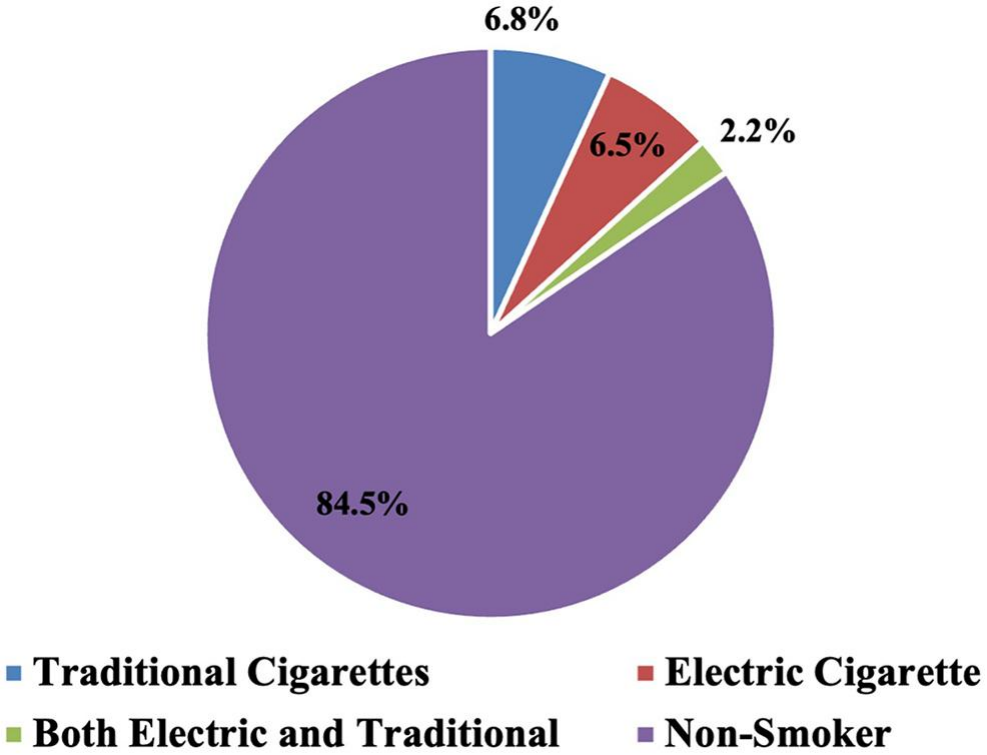
Percent distribution of the studied 278 participants by their smoking status.

Table 2 summarizes the smoking status distribution among the 278 study participants. The overwhelming majority of respondents (84.5%) identified as non-smokers, while a much smaller proportion reported current use of tobacco or e-cigarette products. Specifically, 6.8% were traditional cigarette smokers, 6.5% used electronic cigarettes, and only 2.2% reported dual use of both traditional and electronic cigarettes (Figure 1).

**Table 2:** Distribution of the studied 278 participants by their smoking status.

Smoking status	*n* (%)
Traditional cigarettes	19 (6.8)
Electric cigarette	18 (6.5)
Both electric and traditional	6 (2.2)
Non-smoker	235 (84.5)
Total	278

The findings presented in Table 3 reflect a mixed level of knowledge and perceptions among participants regarding the adverse effects of smoking and e-cigarette use on oral and dental health. While awareness of the potential harms of smoking on oral health was relatively high, with 82.7% recognizing its detrimental effects and specific harm on oral health, specific knowledge about the components of cigarettes (50.0%) and especially e-cigarette liquids (17.6%) was limited. A large majority of participants accurately rejected the notion that e-cigarettes are nicotine-free (99.3%) and safer for oral health (82.4%).

**Table 3:** Participants’ knowledge, beliefs, and perceptions regarding the effects of smoking and e-cigarettes on oral and dental health (*n* = 278).

Question	Yes *n* (%)	No/unsure *n* (%)
Do you know the main components of cigarettes?	139 (50.0)	139 (50.0)
Do you know the main components of e-cigarette liquids?	49 (17.6)	229 (82.4)
Do you believe e-cigarettes are nicotine-free?	2 (0.7)	276 (99.3)
Do you believe e-cigarettes contain less nicotine?	46 (16.5)	232 (83.5)
Are e-cigarettes safer for oral health?	49 (17.6)	229 (82.4)
Are you aware of potential harm to oral health?	230 (82.7)	48 (17.3)
Does smoking affect the mucous membranes?	205 (73.7)	73 (26.3)
Does smoking affect oral tissues?	229 (82.4)	49 (17.6)
Are smokers more prone to oral infections/ulcers?	236 (84.9)	42 (15.1)
Is there a relationship between smoking and oral cancer?	233 (83.8)	45 (16.2)
Does smoking affect sense of taste?	182 (65.5)	96 (34.5)
Does smoking affect sense of smell?	163 (58.6)	115 (41.4)
Does smoking delay oral wound healing?	190 (68.3)	88 (31.7)
Does smoking cause bad breath?	256 (92.1)	22 (7.9)
Does smoking contribute to dry mouth?	222 (79.9)	56 (20.1)
Does smoking increase tooth decay risk?	205 (73.7)	73 (26.3)
Does smoking cause tooth sensitivity?	187 (67.3)	91 (32.7)
Does smoking discolor teeth?	259 (93.2)	19 (6.8)
Does smoking discolor gums over time?	255 (91.7)	23 (8.3)
Does smoking increase plaque/tartar?	209 (75.2)	69 (24.8)
Does smoking cause tooth loss?	185 (66.5)	93 (33.5)
Does smoking affect dental treatment effectiveness?	188 (67.6)	90 (32.4)
Does smoking reduce mouthwash effectiveness?	147 (52.9)	131 (47.1)
Are e-cigarettes more modern/stylish?	72 (25.9)	206 (74.1)
Is there social pressure to choose a type of cigarette?	136 (48.9)	142 (51.1)
Are e-cigarettes a better option for new smokers?	45 (16.2)	233 (83.8)

Most participants acknowledged the association between smoking and various oral health problems, such as bad breath (92.1%), tooth discoloration (93.2%), gum discoloration (91.7%), increased risk of oral cancer (83.8%), and delayed wound healing (68.3%). However, fewer participants (58.6%) recognized smoking's impact on the sense of smell, and only 52.9% believed that smoking could reduce the effectiveness of certain mouthwashes. Interestingly, nearly half of the respondents (48.9%) admitted perceiving social pressure in choosing a type of cigarette, while only 16.2% believed e-cigarettes to be a better option for new smokers.

Table 4 illustrates significant differences in self-reported awareness of the oral and dental health effects of smoking and e-cigarette use between smokers and non-smokers. Non-smokers consistently demonstrated significant higher awareness across multiple areas, including knowledge of cigarette components, the harmful effects of smoking and e-cigarettes on oral health, and their impacts on mucous membranes, oral tissues, infection risk, and oral cancer. Significant disparities also emerged in awareness of smoking's effects on taste, smell, wound healing, and bad breath, as well as on dry mouth, tooth decay, sensitivity, discoloration, plaque buildup, tooth loss, and dental treatment outcomes. Additionally, more non-smokers rejected the idea that e-cigarettes are a safer option for new smokers (*p* = 0.01). However, for several topics, including awareness of e-cigarette liquid components, nicotine content, and social influences on smoking choices, no significant differences were observed between the groups.

**Table 4:** Comparison of awareness of oral and dental health effects between smokers and non-smokers.

Item	Smokers (*n* = 43) yes, *n* (%)	Non-smokers (*n* = 235) yes, *n* (%)	*p* value
Know cigarette components	12 (27.9)	127 (54.0)	0.003*
Know e-cigarette liquid components	11 (25.6)	38 (16.2)	0.20
Believe e-cigarettes are nicotine-free	1 (2.3)	1 (0.4)	0.70
Believe e-cigarettes contain less nicotine	11 (25.6)	35 (14.9)	0.13
E-cigarettes safer for oral health	19 (44.2)	30 (12.8)	<0.0001*
Aware of potential harm to oral health	19 (44.2)	156 (66.4)	0.01*
Affects mucous membranes	14 (32.6)	158 (67.2)	<0.0001*
Affects oral tissues	17 (39.5)	182 (77.4)	<0.0001*
More prone to infections/ulcers	17 (39.5)	182 (77.4)	<0.0001*
Relation to oral cancer	14 (32.6)	178 (75.7)	<0.0001*
Affects sense of taste	16 (37.2)	134 (57.0)	0.03*
Affects sense of smell	12 (27.9)	120 (51.1)	0.01*
Delays wound healing	11 (25.6)	149 (63.4)	<0.0001*
Causes bad breath	16 (37.2)	161 (68.5)	0.0002*
Contributes to dry mouth	18 (41.9)	165 (70.2)	0.001*
Increases tooth decay risk	11 (25.6)	155 (66.0)	<0.0001*
Causes tooth sensitivity	13 (30.2)	148 (63.0)	0.0001*
Discolors teeth	21 (48.8)	167 (71.1)	0.01*
Discolors gums	18 (41.9)	166 (70.6)	0.001*
Increases plaque/tartar	12 (27.9)	149 (63.4)	<0.0001*
Causes tooth loss	13 (30.2)	126 (53.6)	0.01*
Affects dental treatments	13 (30.2)	143 (60.9)	0.0004*
Reduces mouthwash effectiveness	8 (18.6)	119 (50.6)	0.0002*
E-cigarettes more modern	15 (34.9)	57 (24.3)	0.20
Social pressure for cigarette type	16 (37.2)	120 (51.1)	0.13
E-cigarettes better for new smokers	13 (30.2)	32 (13.6)	0.01*

* Significant.

Table 5 presents participants' awareness of the oral and dental health effects of smoking and e-cigarette use by smoking status, revealing significant differences across the four groups. Notably, a higher proportion of e-cigarette users (38.9%) believed e-cigarettes contain less nicotine (*p* = 0.04), and more e-cigarette users (61.1%) and dual users (50.0%) considered them safer for oral health compared to just 12.8% of non-smokers. Non-smokers consistently showed significant higher awareness regarding the harmful oral effects of smoking and vaping, including damage to mucous membranes, oral tissues, risk of infections and ulcers, bad breath, dry mouth, tooth decay, and tooth sensitivity. Similar patterns were seen for awareness of tooth and gum discoloration, plaque buildup, effects on dental treatment outcomes, and reduced effectiveness of mouthwashes. Additionally, a significant difference was observed regarding the perception that e-cigarettes are a better choice for new smokers (*p* = 0.001), with non-smokers largely rejecting this belief. Overall, non-smokers demonstrated the highest levels of awareness across most oral health-related risks.

**Table 5:** Awareness of oral and dental health effects of smoking and e-cigarette use among participants by smoking status (*n* = 278).

Item	Traditional (*n* = 19) yes, *n* (%)	Electronic (*n* = 18) yes, *n* (%)	Both (*n* = 6) yes, *n* (%)	Non-smokers (*n* = 235) yes, *n* (%)	*p* value
Know cigarette components	10 (52.6)	11 (61.1)	4 (66.7)	127 (54.0)	0.56
Know e-cigarette liquid components	3 (15.8)	7 (38.9)	1 (16.7)	38 (16.2)	0.09
Believe e-cigarettes are nicotine-free	1 (5.3)	0 (0.0)	0 (0.0)	1 (0.4)	0.60
Believe e-cigarettes contain less nicotine	3 (15.8)	7 (38.9)	1 (16.7)	35 (14.9)	0.04*
E-cigarettes safer for oral health	5 (26.3)	11 (61.1)	3 (50.0)	30 (12.8)	0.001*
Aware of potential harm to oral health	11 (57.9)	7 (38.9)	1 (16.7)	156 (66.4)	0.02*
Affects mucous membranes	7 (36.8)	10 (55.6)	3 (50.0)	158 (67.2)	0.01*
Affects oral tissues	8 (42.1)	11 (61.1)	2 (33.3)	182 (77.4)	0.01*
More prone to infections/ulcers	10 (52.6)	13 (72.2)	3 (50.0)	182 (77.4)	0.004*
Relation to oral cancer	12 (63.2)	12 (66.7)	3 (50.0)	178 (75.7)	0.08
Affects sense of taste	11 (57.9)	10 (55.6)	4 (66.7)	134 (57.0)	0.89
Affects sense of smell	7 (36.8)	6 (33.3)	3 (50.0)	120 (51.1)	0.14
Delays wound healing	9 (47.4)	8 (44.4)	2 (33.3)	149 (63.4)	0.09
Causes bad breath	9 (47.4)	10 (55.6)	2 (33.3)	161 (68.5)	0.03*
Contributes to dry mouth	7 (36.8)	9 (50.0)	2 (33.3)	165 (70.2)	0.001*
Increases tooth decay risk	6 (31.6)	4 (22.2)	1 (16.7)	155 (66.0)	0.001*
Causes tooth sensitivity	6 (31.6)	4 (22.2)	3 (50.0)	148 (63.0)	0.002*
Discolors teeth	10 (52.6)	7 (38.9)	4 (66.7)	167 (71.1)	0.01*
Discolors gums	8 (42.1)	5 (27.8)	4 (66.7)	166 (70.6)	0.01*
Increases plaque/tartar	5 (26.3)	5 (27.8)	2 (33.3)	149 (63.4)	0.001*
Causes tooth loss	7 (36.8)	6 (33.3)	2 (33.3)	126 (53.6)	0.07
Affects dental treatments	6 (31.6)	7 (38.9)	0 (0.0)	143 (60.9)	0.002*
Reduces mouthwash effectiveness	3 (15.8)	5 (27.8)	0 (0.0)	119 (50.6)	0.003*
E-cigarettes more modern	5 (26.3)	8 (44.4)	2 (33.3)	57 (24.3)	0.25
Social pressure for cigarette type	6 (31.6)	9 (50.0)	1 (16.7)	120 (51.1)	0.09
E-cigarettes better for new smokers	2 (10.5)	10 (55.6)	1 (16.7)	32 (13.6)	0.001*

* Significant.

## 4 Discussion

E-cigarette use has been associated with various oral conditions (23–25). Although maintaining good oral hygiene can help reduce some of the adverse effects of tobacco use, the most effective approach to preserving oral health is to quit smoking entirely and avoid E-cigarette use (26).

In the present study, the prevalence of current e-cigarette use is lower than the rates reported in other studies from Saudi Arabia. For example, Aldhahir et al. (27) found a 32.36% prevalence among 5,012 healthcare students across Saudi universities, while another study reported a 13.5% prevalence among first-year university students in Riyadh (28). These differences suggest variability in vaping trends across different population groups and study contexts within the country. This pattern aligns with global trends, where e-cigarettes are increasingly perceived as a less harmful or more socially acceptable alternative, especially among young adults (29). Additionally, dual users in the study comprised only few participants, indicating a preference for a single product type or possibly representing individuals transitioning between nicotine delivery methods (30).

The levels of awareness observed among the studied population were notably higher than those reported in similar studies conducted in Saudi Arabia (1, 16, 22) and elsewhere (6, 19–21). This suggests that public health messaging in the region has been relatively effective in increasing awareness of the more visible and serious oral health consequences of smoking. Additionally, the findings reflect a growing awareness of the risks associated with e-cigarette use, a particularly important development given the rising popularity of vaping among young adults globally (11). Moreover, the demographic profile of the studied population predominantly young, well-educated, and female participants, likely contributed to the overall high awareness levels, as this group typically has greater health literacy and better access to health-related information through academic and social media platforms (31).

However, participants showed limited knowledge about the contents of tobacco and e-cigarette products. Awareness of less obvious health effects was also low, as only some participants recognized smoking's impact on the sense of smell, and some knew it could reduce the effectiveness of some mouthwashes. This limited awareness is consistent with previous studies (16, 22), which similarly reported poor understanding of the harmful constituents in both conventional and electronic nicotine products. Such gaps are concerning, as knowledge of product content is a crucial factor influencing risk perception and smoking behavior, particularly among youth and young adults (9).

When comparing self-reported awareness of the oral and dental health effects of smoking and e-cigarette use between smokers and non-smokers, non-smokers consistently demonstrated higher awareness across multiple areas. Non-smokers were also more likely to reject the idea that e-cigarettes are a safer option for new smokers. These findings suggest that non-smokers likely, due to greater exposure to health-education messages and lower user-related bias, possess a more accurate understanding of the full scope of oral-health risks associated with tobacco and e-cigarette use (9). In contrast, smokers and e-cigarette users may downplay or remain unaware of these risks, a pattern observed in prior studies where current users often underestimate the harms of their own behaviors (32, 33). This pattern is consistent with a previous multicounty survey of undergraduate dental students from 20 dental schools across 11 countries (6), which similarly reported higher awareness among non-smokers and traditional smokers for most items, while noting that e-cigarette users can show greater awareness of certain specific adverse effects (e.g., dry mouth, black tongue, heart palpitations).

The study findings reveal a concerning trend among e-cigarette users regarding misconceptions about the nicotine content and safety of e-cigarettes for oral health. A significantly greater proportion of e-cigarette users believed that e-cigarettes contain less nicotine compared to other groups, despite growing evidence that many e-cigarette products deliver nicotine levels comparable to, or even exceeding, those of traditional cigarettes (8). This misperception may contribute to the growing appeal of vaping, particularly among younger populations, under the false assumption of reduced harm. Additionally, perceptions of oral health safety varied notably across user groups. Over half of e-cigarette users and half of dual users considered e-cigarettes to be safer for oral health, a belief held by only few of non-smokers. These findings indicate a reduced perception of harm linked to e-cigarette use among smokers, consistent with the results of previous studies. King et al. (32) and Hughes et al. (34) both reported lower harm perceptions among male smokers in studies involving American and British youth populations. Similarly, regional studies conducted in Saudi Arabia have documented comparable patterns of risk misperception among smokers (35). Recent findings among young adults in Saudi Arabia provide further evidence of ongoing misperceptions surrounding the safety profile and nicotine content of e-cigarettes, underscoring the need for improved public health messaging (36). Moreover, recent evidence from young adult populations confirms the persistence of misperceptions regarding e-cigarettes and oral health despite growing research interest (7, 37). All these findings indicate that users of these products are more likely to underestimate their health risks, likely influenced by marketing messages and social narratives framing e-cigarettes as a healthier alternative to traditional smoking (13).

A key strength of this study is that it is one of the first conducted in the Al-Madinah region to assess public awareness of the adverse oral and dental health effects linked to both tobacco and e-cigarette use. Recent literature in 2025 further underscores the need for proactive oral-health interventions targeting e-cigarette users (7, 37). By including a range of user categories, traditional smokers, e-cigarette users, dual users, and non-smokers, the study enabled meaningful comparisons across different patterns of smoking behavior. Furthermore, a relatively large sample size was obtained through online distribution, enabling access to diverse demographic groups and addressing an increasingly relevant public health issue given the rising popularity of e-cigarettes in the region.

However, certain limitations should be acknowledged. This study relied on an online, non-probability convenience sampling method, which may have introduced selection bias and limited the representativeness of the findings. The study also depended on self-reported data, which is subject to recall and social desirability bias. Furthermore, the sample was predominantly young, female, and highly educated, which may restrict the generalizability of the results to other segments of the Saudi population, particularly older or less educated individuals. The small sizes of certain subgroups limit the statistical stability of their respective estimates; these results should therefore be considered preliminary and descriptive. Future research should include a more balanced demographic to ensure broader representation and more generalizable results. The cross-sectional design captures awareness at a single point in time and does not establish causal relationships. Additionally, the online distribution method may have excluded individuals with limited internet access or lower digital literacy.

## 5 Conclusion

This study highlights significant gaps in awareness concerning the oral health risks of e-cigarette use compared to traditional smoking. While awareness was relatively higher among non-smokers and traditional smokers, misconceptions about the safety of e-cigarettes persisted among e-cigarette and dual users. These findings underscore the urgent need for targeted public health initiatives that dismantle misconceptions by delivering tailored risk messages to key groups like young and dual users through digital and campus channels, while simultaneously leveraging dental consultations in Madinah for direct patient education.

## Data Availability

The raw data supporting the conclusions of this article will be made available by the authors, without undue reservation.
